# Impact of Menopausal Status and Female Reproductive Aging on the Outcomes of Bariatric Surgery: A Narrative Review

**DOI:** 10.7759/cureus.103873

**Published:** 2026-02-18

**Authors:** Alexis C Spencer-Vargas, Harvey N Mayrovitz

**Affiliations:** 1 Medical School, Nova Southeastern University Dr. Kiran C. Patel College of Osteopathic Medicine, Davie, USA; 2 Medical Education, Nova Southeastern University Dr. Kiran C. Patel College of Allopathic Medicine, Davie, USA

**Keywords:** bariatric surgery, body composition, complications, female reproductive aging, menopause, metabolic surgery, skeletal health, weight loss outcomes, women’s health

## Abstract

Women comprise the majority of patients undergoing metabolic and bariatric surgeries, yet the influence of menopausal status and female reproductive aging on postoperative outcomes remains poorly understood. The menopausal transition is associated with profound hormonal, metabolic, and skeletal changes that may modify the efficacy and risk profile of bariatric surgery. This narrative review aims to synthesize current evidence on how menopausal status influences bariatric surgery outcomes, including weight loss, metabolic outcomes, body composition, and skeletal health.

A literature search was conducted using PubMed/MEDLINE, Embase, and Web of Science to identify peer-reviewed studies evaluating bariatric surgery outcomes in adult women stratified by menopausal status, reproductive aging stage, or age-based proxies for menopause. Available evidence suggests that postmenopausal women experience less weight loss after bariatric surgery than premenopausal women but achieve comparable reductions in visceral adiposity and improved lipid profiles. Body composition analyses indicate that bariatric surgery does not uniformly exacerbate sarcopenia in postmenopausal women; however, disproportionate loss of fat-free mass relative to total weight loss in middle-aged and older patients is associated with adverse cardiometabolic outcomes. Skeletal health is the most negatively affected outcome, with postmenopausal women demonstrating accelerated declines in bone mineral density (BMD), deterioration of bone microarchitecture, and persistent elevations in bone turnover markers following both laparoscopic sleeve gastrectomy (LSG) and Roux-en-Y gastric bypass (RYGB).

This review shows that menopausal status is an important yet underrecognized modifier of bariatric surgery outcomes in women. While bariatric surgery confers metabolic benefits in postmenopausal patients, it amplifies skeletal vulnerability in this hormonally susceptible population. These findings highlight the need for menopause-informed preoperative counseling, postoperative monitoring, and targeted strategies to mitigate bone loss. Future studies should prioritize large, longitudinal, menopause-stratified cohorts incorporating hormonal, metabolic, body composition, and skeletal assessments to better inform clinical decision-making.

## Introduction and background

In 2021, an estimated 1.11 billion women were classified as being either overweight or obese, comprising nearly half of all adult females globally [1]. The prevalence of obesity in women has more than doubled, rising from 10.2% in 1990 to 20.8% in 2021 [1]. Among adult women in the United States, obesity prevalence reaches a peak of 48.7% in women aged 50-54 years, after which it declines after 75 years [2]. Obesity in women is associated with increased risk of type 2 diabetes mellitus, hypertension, dyslipidemia, metabolic syndrome, obstructive sleep apnea [3], osteoarthritis, infertility, depression, and various cancers [4]. Importantly, this peak in obesity prevalence coincides with the typical age range of the menopausal transition [5], suggesting a potential interaction between reproductive aging and obesity-related health risk.

The menopausal transition refers to the period leading up to and immediately following the last menstrual period, lasting 1-3 years on average, and occurring in 90% of women between ages 45 and 56 [5]. In the United States, the average reported age is 51.4 years [6,7]. During this period, women experience a shift from a gynoid to an android fat distribution, with a 5.54% annual increase in body fat [8]. This transition begins approximately two years before the last menstrual period in the premenopausal stage, when the rate of fat gain doubles and lean mass declines, and continues for two years after the final menstrual cycle [9].

**Figure 1:**
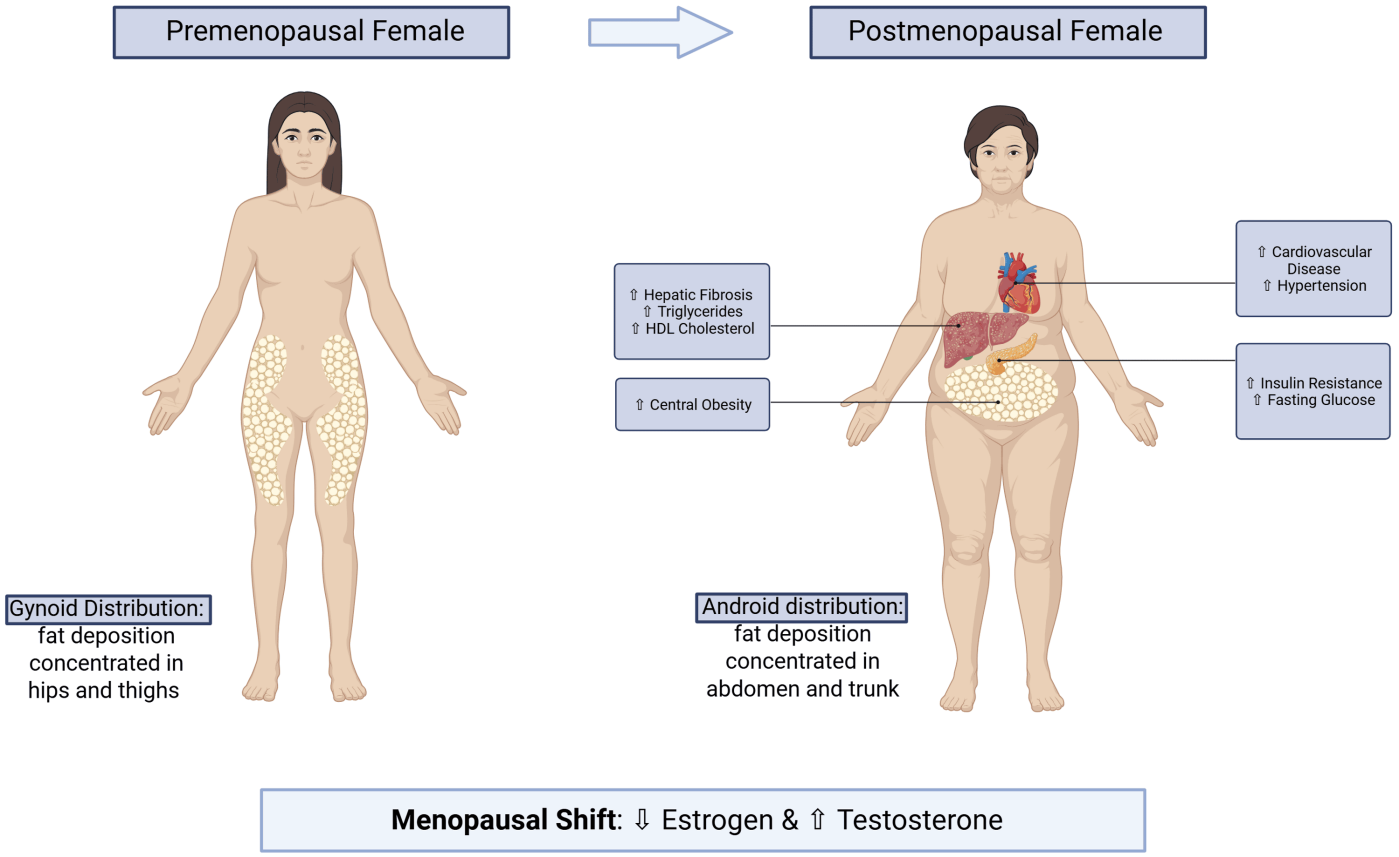
Physiological and metabolic changes associated with the menopausal shift Illustration depicting the physiological shift in fat distribution that occurs during menopause. Premenopausal women typically exhibit a gynoid pattern of fat deposition concentrated in the hips and thighs, whereas menopause is associated with redistribution toward an android pattern characterized by increased abdominal adiposity. Created in BioRender (available at https://BioRender.com/e62fl45).

Gynoid fat distribution is one where fat accumulates in the hips, buttocks, and thighs, whereas android fat distribution is in the abdomen and trunk, as demonstrated in Figure 1 [10]. This change is primarily driven by declining estrogen and increasing androgen production, which promote conversion to an androgenic body habitus and exacerbate adverse metabolic changes, including increased risk of insulin resistance [11], cardiovascular disease [12], and hepatic fibrosis [13]. After menopause, females have higher odds of all metabolic syndrome components, including fasting glucose, hypertension, elevated triglycerides, low high-density lipoprotein (HDL) cholesterol, and central obesity [14,15]. These physiological changes highlight menopause as a biologically distinct period that may modify the response to obesity interventions.

Bariatric surgery is currently the most effective weight loss therapy, indicated in individuals with a body mass index (BMI) over 40 kg/m2 or with a BMI between 35 and 40 kg/m2 with obesity-related comorbidities [16,17]. Although recent pharmacologic advances, particularly glucagon-like peptide-1 (GLP-1) receptor agonists, have demonstrated promising short-term weight loss, bariatric surgery remains the most effective and durable intervention, with greater long-term weight reduction and lower risk of weight regain following treatment discontinuation [18].

The terms bariatric and metabolic surgery refer to the same procedures but differ in their primary treatment goals. Bariatric surgery historically refers to gastrointestinal operations to induce weight loss in patients with obesity, whereas metabolic surgery refers to the same procedures but with a primary intent to improve metabolic status, particularly in type 2 diabetes, dyslipidemia, and cardiovascular disease [19,20]. Metabolic surgery also differs in that it is typically offered to patients with a BMI as low as 30 kg/m2 [21,22].

There are five primary bariatric procedures performed in the United States, with laparoscopic sleeve gastrectomy (LSG) and Roux-en-Y gastric bypass (RYGB) accounting for greater than 90% of cases [23]. LSG is the most commonly performed procedure and involves the removal of approximately 70-85% of the stomach, resulting in accelerated gastric emptying [23]. RYGB is the second most common procedure and entails the creation of a small gastric pouch that is surgically connected to the jejunum [23].

Less commonly performed procedures include adjustable gastric banding (AGB), biliopancreatic diversion with or without duodenal switch (BPD/BPD-DS), and single anastomosis duodeno-ileal switch (SADI-S). AGB is a procedure in which an inflatable band is placed around the upper portion of the stomach to create a small, adjustable pouch [24]. BPD involves the removal of the pylorus of the stomach from the duodenum, and the distal ileum is subsequently attached to the stomach, while BPD-DS is a similar procedure but preserves the pylorus [25]. These procedures are now rarely used due to higher malabsorptive complications and nutritional risk profiles [26]. SADI-S is a simplified alternative to BPD-DS that combines a sleeve gastrectomy with a single intestinal bypass designed to mitigate the malabsorptive complications of BPD/BOD-DS [27].

Historically, women comprise the majority of patients undergoing bariatric surgery at around 75-80% of the procedures performed [28,29]. Despite its effectiveness and low complication rate, bariatric surgery utilization remains extremely low amongst women in the United States, with only 1-2% of individuals who are eligible receiving this surgery [30]. Among females who do undergo bariatric surgery, the mean age is approximately 44 years [31], reflecting an increasing trend toward midlife surgery. The average age of bariatric surgery recipients has risen from 38.9 years in 1993 to 44.4 years in 2016 [31]. As a result, an increasing proportion of women undergoing metabolic and bariatric surgery are in the peri- or postmenopausal period, underscoring the clinical relevance of examining how reproductive aging may influence surgical outcomes.

Given the hormonal and metabolic changes that accompany the menopausal transition, reproductive aging may be an important yet underexplored factor influencing the efficacy of bariatric surgery in women. Despite increasing numbers of women undergoing bariatric surgery during midlife and beyond, few studies have directly examined whether outcomes differ between pre- and post-menopausal women. Therefore, the purpose of this narrative review is to synthesize current evidence on how menopausal status and female reproductive aging influence weight-loss outcomes, metabolic improvements, body composition changes, and related health parameters following bariatric surgery.

## Review

Materials and methods 

Study Design

This study is a narrative review examining the influence of menopausal status and female reproductive aging on outcomes following metabolic and bariatric surgery. A narrative approach was chosen because of heterogeneity in study design, outcome measures, and definitions of menopausal status, which limits the feasibility of a quantitative synthesis. Accordingly, no quantitative synthesis, meta-analysis, or statistical pooling was performed. 

Literature Search 

A literature search was conducted using PubMed/MEDLINE, Embase, and Web of Science. In PubMed/MEDLINE, searches were performed using combinations of keywords related to bariatric surgery, menopausal status, and postoperative outcomes, including bariatric surgery, metabolic surgery, Roux-en-Y gastric bypass, sleeve gastrectomy, menopause, postmenopausal, age, reproductive aging, and weight-loss outcome terms. The PubMed search strategy was based on the following title and abstract search: (“bariatric surgery” OR “metabolic surgery” OR “Roux-en-Y gastric bypass” OR “sleeve gastrectomy” OR “single-anastomosis duodeno-ileal switch”) AND (“excess weight loss” OR “%EWL” OR “total weight loss” OR “%TWL” OR “BMI change” OR “weight regain”) AND (women OR female AND (menopause OR postmenopausal OR age OR aging OR “older women”), with filters applied for full text, human studies, female sex, English language, and adult and middle-aged populations. Comparable search strategies and filters were adapted for Embase and Web of Science. Reference lists of relevant articles were also manually screened to identify additional studies of interest. 

Eligibility Criteria 

Studies were included if they involved adult women undergoing metabolic or bariatric surgery and reported postoperative outcomes stratified by menopausal status, reproductive aging stage, or age as a proxy for menopausal status. Eligible outcomes included total weight loss percentage, excess weight loss percentage, BMI change, metabolic syndrome components, glycemic control, fracture risk, lean mass, and visceral adiposity. Only peer-reviewed, full-length articles published in English were considered. Studies focused exclusively on pediatric populations, pregnancy-related outcomes, male-only cohorts, revisional procedures, as well as editorials, letters, conference abstracts, and other non-peer-reviewed publications, were excluded. 

Definition of Menopausal Status 

Menopausal status was defined by using hormone status or menstrual history to classify patients as premenopausal or postmenopausal when available. When reproductive data were unavailable, age-based proxies were used. Women aged 20-45 years were considered premenopausal, and those aged 55-65 years were considered postmenopausal. Women aged 46-54 years were not assigned to menopausal categories because this age range encompasses substantial biological heterogeneity, including late premenopausal, perimenopausal, and early postmenopausal states, which would increase the risk of misclassification and reduce the interpretability of comparisons.* *

Data Synthesis 

Given the methodological variability across studies, results were synthesized qualitatively, with emphasis on identifying consistent patterns, procedure-specific differences, and knowledge gaps related to reproductive aging and surgical outcomes.

Results

Weight Loss Outcomes 

In a study of 614 female patients divided into premenopausal (n=545) and postmenopausal status (n=69), there was a statistically significant difference in weight loss outcomes after bariatric surgery [32]. Postmenopausal women achieved significantly less percent excess weight loss (%EWL) and percent total weight loss (%TWL) compared to premenopausal women [32]. Specifically, mean %EWL was 87.8% in premenopausal women versus 73.8% in postmenopausal women (p < 0.01), while %TWL was 38.8% and 35.9%, respectively (p < 0.01) [32]. Absolute weight loss also differed between groups, with premenopausal women losing an average of 50 kg compared with 43 kg in postmenopausal women (p < 0.001) [32].

In studies where menopausal status was not directly reported, age was used as a parameter. For example, in one study, a sex-specific analysis was used to stratify women into premenopausal (<45 years of age, n = 1,199) or postmenopausal (≥ 55 years of age n=157) [33]. After adjustments for procedure type and baseline percent excess body weight (%EBW), premenopausal women achieved significantly greater %EBW loss (%EBWL) than postmenopausal women at both 12 and 24 months following bariatric surgery (overall F₁,₁₃₅₅ = 6.9, p = 0.001; both time points p < 0.0005) [33]. There were no significant differences when comparing other age groups, such as 20-25 vs. 30-35, 30-35 vs. 40-45 years, or 20-25 vs 40-45 years (all p values >0.2) [33]. In women, age became a predictor of %EBWL only at older ages, whereas among men (n=289), age did not predict %EBWL at either 12- or 24-months post-surgery (both p values >0.3) [33].

Additionally, this study suggests that surgical procedure type may contribute to the observed menopausal differences among women. As compared to postmenopausal women, premenopausal women experienced significantly greater %EBWL after AGB at both 12 and 24 months, translating to approximately 7 kg greater weight loss at 24 months (overall F_1,323_ = 4.2, p = 0.016) [33]. In contrast, no significant differences in weight loss were detected between these groups following RYGB (overall p = 0.24) [33].

*Metabolic and Body Composition Outcomes* 

Bariatric surgery significantly improves metabolic outcomes postoperatively in patients by improving gut hormone signaling, bile acid metabolism, urinary sodium excretion, insulin resistance, and insulin production [34].

In a group of (n=1,234) postmenopausal women who had RYGB, long-term observational studies demonstrated statistically significant reductions in visceral adipose tissue (VAT), total fat mass percentage (total FM%), android fat, and improved lipid profile compared with non-operated women (n=1,475) matched for age and BMI using propensity score analysis [35]. Post-RYGB women exhibited significantly lower total FM% (39.4 ± 8.4% vs 45.9 ± 5.4%, p < 0.01), VAT (751 ± 496 g vs 1,295 ± 688 g, p < 0.001), and android fat (40.8 ± 12.7% vs 51.5 ± 9.0%, p < 0.001) compared with age and BMI matched non-operated controls [35]. These changes were accompanied by a higher lean mass (LM) percentage (57.7 ± 8.0% vs 52.5 ± 5.0%,p < 0.001) and an improved lipid profile, including lower total cholesterol (4.8 ± 0.9 vs 5.5 ± 0.9 mmol/L, p < 0.001), lower LDL cholesterol (2.4 ± 0.8 vs 3.4 ± 0.8 mmol/L, p < 0.001), and higher HDL cholesterol (1.9 ± 0.4 vs 1.6 ± 0.4 mmol/L, p = 0.008) [35].

Preservation of lean mass in this study is clinically relevant, as evidence suggests that lean mass is inversely associated with cardiovascular risk [36] and it contributes to the long-term preservation of body weight [37]. In a large cohort study of 5,889 individuals who underwent RYGB (84%) or LSG (16%), it was found that patients ≥ 45 years with a high TWL or a ratio of fat-free mass loss relative to total weight loss (FFML/WL) ≥ 30% (fully adjusted hazard ratio (HR) = 1.68 (1.03-2.75) and HR = 1.78 (1.12-2.85), respectively) had an increased risk of major adverse cardiovascular events (MACE) [38].

Skeletal Outcomes 

One adverse outcome of bariatric surgery is a decrease in bone health, with several studies showing that bone mineral density (BMD) decreases significantly in the years following surgery [39-41]. Given that postmenopausal women already face adverse skeletal changes due to a decrease in estrogen [42], it is important to examine the outcomes of bone health in this patient population following bariatric surgery. 

Changes in Bone Density, Skeletal Microarchitecture, and Bone Strength: In a study of postmenopausal women (n=15), premenopausal women (n=25), and men (n=13), LSG was associated with a significant decrease in femoral neck areal bone mineral density (aBMD) and total hip aBMD at 12 months post operatively (6.7% and 8.0%, respectively; p<0.01 for both) [43]. Declines in lumbar spine aBMD were also significant, with postmenopausal women experiencing a greater decline compared to premenopausal women or men (p = 0.04) [43].

Menopausal differences were more pronounced at peripheral skeletal sites following LSG, where postmenopausal women experienced greater losses in total volumetric BMD (vBMD) at the radius (-2.4% in postmenopausal vs + 0.6% in premenopausal ), total vBMD at the tibia (-5.8% vs -2.4 vs -1.1% in men), and cortical vBMD at the tibia (-3.3% in vs -0.5% vs + 0.4%) (all p<0.05) [43]. These structural changes were accompanied by greater declines in estimated bone strength among postmenopausal women, including a 5.7% reduction in predicted tibial failure load compared with 1.7% in premenopausal women and no decline in men (p<0.05) [43].

Similar adverse skeletal patterns were observed following RYGB in a cohort of premenopausal women (n=27), postmenopausal women (n=11), and men (n=10), where postmenopausal women demonstrated greater increases in bone turnover marker C-terminal telopeptide (CTx +338% vs. +260%, p = 0.049) and more pronounced declines in total hip aBMD, spinal vBMD, and tibial vBMD over 12 months compared with premenopausal women and men (all p≤0.02) [44].

Consistent with these findings, a study comparing women undergoing LSG (n=33) or RYGB (n =33) reported similar postoperative declines in BMD and determined that menopausal status, along with postoperative lean mass, are the main predictors of BMD, independent of surgical technique [45].

Long-Term Skeletal Outcomes in Postmenopausal Women: Evidence of increased incidence of adverse skeletal outcomes in postmenopausal women is further supported by long-term follow-up data. In a prospective study of 59 obese women (mean age 46 ± 8 years) followed for three years after RYGB surgery, it was found that BMD continued to significantly decrease between the first and third postoperative years, particularly at the lumbar spine and femoral neck [46]. After stratification by menopausal status, it was found that menopausal women (n=13) had approximately double the reductions in total hip and lumbar spine BMD over three years compared to premenopausal women (n=46), resulting in significantly lower femoral neck (0.87 ± 0.06 vs. 0.98 ± 0.11) and lumbar spine BMD (0.94 ± 0.15 vs. 1.06 ± 0.13) at three years post-RYGB (all p<0.05) [46]. Although Z scores of these patients were found to be higher than those of women of the same age, indicating residual protection, they still demonstrate a greater rate of bone loss and deterioration in bone microarchitecture relative to premenopausal women and men [46]. 

Bone Turnover and Secondary Hyperparathyroidism: Not all studies demonstrate uniform reductions in aBMD at axial skeletal sites following bariatric surgery among postmenopausal women. In a study of 13 women (seven pre- and six postmenopausal) examined 1-5 years after RYGB, no significant differences in lumbar spine or femoral neck BMD were observed compared with age- and BMI-matched non-operated controls, despite evidence of increased skeletal resorption [47]. Parathyroid hormone (PTH) levels were approximately threefold higher in the RYGB group compared with controls at both baseline (10.2 ± 6.0 vs. 3.4 ± 1.0 pM) and follow-up after 6 months of dietary supplementation (9.1 ± 5.5 vs. 3.2 ± 1.3 pM), consistent with ongoing secondary hyperparathyroidism [47]. 

These patterns are further supported by a separate cohort of 26 postmenopausal women evaluated 1-5 years after RYGB, demonstrating that bone resorption remained elevated, with CTx levels 65% higher than in age- and BMI-matched controls (0.71 ± 0.21 vs. 0.43 ± 0.15 ng/mL; p<0.01), accompanied by higher parathyroid hormone levels (68.3 ± 35 vs. 49.4 ± 16 pg/mL; p=0.02) [48]. Together, these studies suggest that secondary hyperparathyroidism and increased bone resorption may persist long after surgery and primarily affect cortical bone sites, such as the femoral neck and radius, while relatively sparing trabecular-rich regions, such as the lumbar spine. This dissociation between biochemical markers of bone turnover and areal BMD measurements may help explain the heterogeneity of axial BMD findings across studies and underscores the limitation of DXA alone in fully capturing postoperative skeletal risk in postmenopausal women.

A comparative summary of primary weight loss and skeletal outcomes following bariatric surgery in premenopausal and postmenopausal women is presented in Table 1. 

**Table 1:** Key bariatric surgery outcomes by menopausal status ↑ = increase; ↓ = decrease; ↓↓/↑↑ = significant change relative to premenopausal women; aBMD = areal bone mineral density; vBMD = volumetric bone mineral density; CTx = C-terminal telopeptide

Outcome	Premenopausal Women	Postmenopausal Women
Weight Loss Outcomes		
% Excess Weight Loss (%EWL)	↑↑	↑
% Total Weight Loss (%TWL)	↑↑	↑
Average weight loss	↑↑	↑
Skeletal Outcomes		
Femoral neck aBMD	↓	↓↓
Lumbar spine aBMD	↓	↓↓
Radius vBMD loss	↓	↓↓
Tibia vBMD total	↓	↓↓
Tibia vBMD cortical	↓	↓↓
Bone turnover markers (CTx)	↑	↑↑

Discussion 

The objective of this narrative review was to synthesize current evidence on how menopausal status and female reproductive aging influence weight-loss outcomes, metabolic improvements, body composition changes, and related health parameters following bariatric surgery. This review highlights that menopausal status modifies the efficacy of bariatric surgery across different outcomes. While there are marked differences in surgical outcomes between pre- and postmenopausal women, bariatric surgery still confers benefit to obese postmenopausal women. 

Studies are consistent in reporting that postmenopausal women tend to achieve less total weight loss and excess weight loss following bariatric surgery compared with premenopausal women; however, many of these findings are derived from retrospective analyses, and the extent to which baseline differences and confounding variables were controlled varies across studies. Declining rates of estrogen in menopause are associated with downregulation of genes involved in beta oxidation, an increase in lipid synthesis, and a decrease in energy expenditure, which all contribute toward visceral fat accumulation [49,50]. These factors should be considered when counseling postmenopausal patients on the expected efficacy of gastric bypass surgery.

While postmenopausal women tend to achieve lower weight loss than premenopausal women and men, studies demonstrate sustained reductions in visceral adiposity and favorable lipid profiles. This finding is particularly important given that menopause induces an increase in visceral adiposity, which triggers enhanced lipolysis, producing excessive free fatty acids that contribute to insulin resistance [49]. Therefore, bariatric surgery in postmenopausal women may help counteract these metabolic shifts by reducing visceral adiposity.

Body composition after bariatric surgery is an important determinant of cardiometabolic risk, particularly in middle-aged and older patients. Evidence indicates that lean mass can be preserved in postmenopausal women following bariatric surgery, and surgery itself does not appear to inherently induce sarcopenia in this population. However, studies also demonstrate that when disproportionate loss of fat-free mass relative to total weight loss does occur, it is associated with adverse cardiovascular outcomes in individuals aged 45 years and older. Given that estrogen decline during menopause is associated with age-related changes in muscle mass and muscle quality [51], preservation of lean mass may be especially relevant for risk stratification and long-term cardiometabolic outcomes in postmenopausal women undergoing bariatric surgery.

Skeletal health is by far the most negatively affected outcome of bariatric surgery in postmenopausal women. Multiple studies demonstrate that postmenopausal women experience accelerated declines in bone mineral density across multiple sites, deterioration in bone microarchitecture, and decreased bone strength post-bariatric surgery. Estrogen deficiency in menopausal women is known to increase osteoclast differentiation and activation, thus accelerating bone loss [42]. This makes postmenopausal women considering bariatric surgery particularly susceptible to an increased likelihood of fragility fracture post-surgery.

Another interesting finding is that the adverse skeletal effects observed in postmenopausal women appear independent of surgical technique [45]. Both LSG and RYGB are associated with declines in areal and volumetric BMD, as well as similar patterns of microarchitectural deterioration [43-45], suggesting that factors independent of malabsorption may play a role. Shared factors, including postoperative changes in body composition and altered PTH homeostasis, likely contribute substantially to skeletal decline [44,47,48]. This is further supported by studies demonstrating persistent elevations in bone resorption and secondary hyperparathyroidism years after surgery [47,48], even in cases where aBMD at sites such as the lumbar spine appears preserved [47]. Together, these observations suggest that dual-energy X-ray absorptiometry (DXA)-based BMD measurements may underestimate skeletal risk in postmenopausal women after bariatric surgery, particularly in the presence of persistently elevated biochemical markers of bone turnover [44,47,48].

These findings suggest that while bariatric surgery confers substantial metabolic and cardiometabolic benefits, it simultaneously amplifies skeletal fragility in an already hormonally vulnerable population. Therefore, bone-protective strategies and close monitoring of resorptive bone markers should be implemented in the postmenopausal population.

The findings summarized in this review have important clinical implications for the management of obesity in women undergoing bariatric surgery. Although bariatric surgery remains the most effective treatment for obesity, outcome expectations should be considered within a reproductive aging framework when counseling female patients. Menopausal status should be considered when interpreting surgical efficacy, as postmenopausal women may experience distinct outcome profiles. Greater attention should be directed toward maintaining skeletal health in postmenopausal women, who appear more susceptible to bone microarchitectural deterioration following surgery, including through regular postoperative assessment of bone mineral density. Together, these findings support the need for individualized preoperative counseling, proactive monitoring and intervention of bone loss after surgery, and the establishment of realistic, patient-centered goals based on reproductive age status.

This narrative review has several limitations. First, the narrative design and heterogeneity of included studies prevent quantitative synthesis. Although there were included studies that classified participants as premenopausal or postmenopausal, there was no standardized criterion based on hormonal measurements. Menopausal status was primarily determined by menstrual history, typically defined by the timing of the final menstrual period. In one study, not stratified by menopausal status, age-based proxies were required, which may introduce misclassification. To minimize this risk, women aged 46-54 years were excluded from proxy-based analyses; however, this approach may limit generalizability to women undergoing the perimenopausal transition.

Additionally, studies that stratified outcomes by menopausal status often had small sample sizes, which limits the strength of the observed patterns. Data on newer bariatric procedures, including SADI-S, were notably lacking, further limiting procedure-specific conclusions. Despite these limitations, consistent patterns emerged across outcomes, supporting the relevance of menopausal status as an important modifier of bariatric surgery outcomes in women. 

Despite women comprising the vast majority of bariatric patients, significant gaps remain in understanding how menopausal status and female reproductive aging influence postoperative bariatric surgery outcomes, particularly for newer metabolic procedures. No studies were identified evaluating SADI-S across stages of female reproductive aging. Given the growing clinical interest in SADI-S and its distinct metabolic and malabsorptive profile compared with more commonly performed procedures, the absence of menopause-stratified outcome data represents an important gap in the literature. Future studies should evaluate weight loss efficacy, metabolic outcomes, nutritional risk, and skeletal health following SADI-S across stages of female reproductive aging. 

Additionally, only a limited number of studies directly stratify outcomes by menopausal status, requiring the use of age-based proxies when reproductive or hormonal data are unavailable. Women aged 46-54 years were intentionally excluded from proxy-based analyses because this age range encompasses substantial biological heterogeneity, including late premenopausal, perimenopausal, and early postmenopausal states. Although this approach reduces the risk of misclassification, it may limit the generalizability of findings to women undergoing the perimenopausal transition, a period marked by variable hormonal and metabolic fluctuations. Consequently, the conclusions of this review are most applicable to clearly premenopausal and postmenopausal populations rather than women in the menopausal transition. 

Among studies that do stratify by menopausal status, sample sizes are often small, limiting the strength of conclusions. Additionally, there are few longitudinal designs that track changes in outcomes across the hormonal transitions that are characteristic of female reproductive aging. Future research should prioritize larger, longitudinal, menopause-stratified cohorts incorporating direct assessments of hormonal status alongside metabolic, body composition, and skeletal measurements to better characterize the interaction between menopausal status and bariatric surgery outcomes.

## Conclusions

Menopausal status and female reproductive aging are important yet underrecognized modifiers of bariatric surgery outcomes in women. While bariatric surgery confers metabolic and cardiometabolic benefits in postmenopausal patients, skeletal health emerges as the most vulnerable outcome, with evidence of accelerated bone loss, microarchitectural deterioration, and significant elevations in bone turnover markers that persist years after surgery.

Incorporating menopausal status into preoperative counseling, postoperative monitoring, and long-term management may help inform risk stratification and patient-centered decision-making. Future research should prioritize large, longitudinal, menopause-stratified cohorts with integrated assessments of hormonal status, body composition, metabolic health, and skeletal outcomes to better define the long-term risks and benefits of bariatric surgery across the stages of female reproductive aging.
